# Improved salivary cortisol rhythm with dual-release hydrocortisone

**DOI:** 10.1530/EC-18-0257

**Published:** 2018-07-24

**Authors:** Filippo Ceccato, Elisa Selmin, Chiara Sabbadin, Miriam Dalla Costa, Giorgia Antonelli, Mario Plebani, Mattia Barbot, Corrado Betterle, Marco Boscaro, Carla Scaroni

**Affiliations:** 1 Endocrinology UnitDepartment of Medicine DIMED, University-Hospital of Padova, Padova, Italy; 2 Laboratory MedicineDepartment of Medicine DIMED, University-Hospital of Padova, Padova, Italy

**Keywords:** salivary cortisol, adrenal insufficiency, glucocorticoid therapy, cortisol rhythm, dual-release hydrocortisone

## Abstract

**Introduction and Aim:**

The purpose of replacement therapy in adrenal insufficiency (AI) is mimicking endogenous cortisol levels as closely as possible: dual release hydrocortisone (DR-HC) has been introduced to replicate the circadian cortisol rhythm. Multiple daily saliva collections could be used to assess the cortisol rhythm during real life: our aim was to study the salivary cortisol profile in AI.

**Materials and Methods:**

We prospectively evaluated, in an observational study, 18 adult outpatients with AI (11 primary and 7 secondary AI), switched from conventional treatment (conv-HC, 25 mg/day) to the same dose of DR-HC. We collected six samples of saliva in a day, measuring cortisol (F) and cortisone (E) with LC-MS/MS. Forty-three matched healthy subjects served as controls.

**Results:**

F levels were similar in the morning (and higher than controls) in patients treated with conv-HC or DR-HC; otherwise F levels and exposure were lower in the afternoon and evening in patients with DR-HC, achieving a cortisol profile closer to healthy controls. Daily cortisol exposure, measured with area under the curve, was lower with DR-HC. Morning F and E presented sensitivity and specificity >90% to diagnose AI (respectively threshold of 3 and 9.45 nmol/L). Total cholesterol and HbA1c levels reduced with DR-HC.

**Conclusions:**

Salivary cortisol daily curve could be used as a new tool to assess the cortisol profiles in patients treated with conv-HC and DR-HC. A lower daily cortisol exposure was achieved with DR-HC (despite the same HC dose), especially in the afternoon-evening.

## Introduction

Adrenal insufficiency (AI) is a rare endocrine disorder characterized by insufficient cortisol production, due to impaired ACTH or cortisol secretion, depicting secondary or primary AI (respectively SAI and PAI) (1, 2, 3). The most common cause of acquired and persistent SAI in adults is a result of a pituitary tumor: its mass effect or pituitary-directed treatments (surgery/radiotherapy) may impair hypothalamic–pituitary–adrenal (HPA) axis function (3, 4). Furthermore, the clinical picture of SAI may be complicated by the association of other pituitary deficiencies, which could affect both HPA axis and glucocorticoid (GC) treatment (5). A more severe degree of AI could characterize PAI, due to the impairment of both cortisol and aldosterone production: patients with PAI usually need larger doses of GC treatment combined with mineralocorticoid (2, 6).

The conventional GC formulations are hydrocortisone (HC) and cortisone acetate (CA), and they are administered in two or three divided doses (the higher in the morning), as suggested in recent Consensus and Guidelines (2, 6). Nevertheless, none of the proposed regimen is currently able to replicate the physiological cortisol circadian rhythm, characterized by a concentration peak in the morning and a nadir during the evening (7). Therefore, it is not uncommon under- or over-exposure to GC treatment: the former induces these patients to the risk of adrenal crisis and fatigue with impaired quality of life; in the latter, the AI patients are prone to develop metabolic and cardiovascular consequences as those observed in Cushing’s syndrome (CS) (7, 8, 9).

Recently, dual-release HC (DR-HC) has been introduced: it consists in an immediate releasing coat and in an extended-release core, providing a peak of GC during the morning followed by a gradual decrease during the day. Cortisol exposure in terms of area under the curve (AUC) with DR-HC is 20% lower to that obtained with conventional-HC (conv-HC) (10, 11, 12).

AI treatment is further complicated by the absence of a universally accepted method to objectively evaluate the adequacy of GC substitutive therapy. We had recently proposed multiple salivary cortisol (F) collections to assess the adequacy of conv-HC in AI (13, 14). Furthermore, recently some authors reported that cortisone (E) measurement in saliva could be used to exclude GC contamination (15, 16).

Therefore, our aim was to study the F and E rhythm with multiple salivary collections in AI patients treated with conv-HC and then switched to DR-HC.

## Materials and methods

### Patients

We prospectively evaluated, in an observational study, 18 adult AI outpatients (11 PAI and 7 SAI, 6 males and 12 females, mean age 44 ± 12 years). AI was suspected clinically and confirmed biochemically with basal serum morning cortisol <138 nmol/L (before GC treatment) or inadequate cortisol response after cosyntropin test (2, 3).

Patients were on a stable conv-HC dose with HC (*n* = 11) or CA (*n* = 7) for at least 1 year before baseline visit. In Italy, CA is more easily available than HC: the former can be bought with a general practitioner’s prescription in any pharmacy, while the latter is distributed after import only in reference hospitals with an endocrinologist’s prescription. Therefore, CA is routinely preferred if patients live far from a dispensing hospital. Thus, to analyze different treatment properly, we considered HC equivalent doses (20 mg HC = 25 mg CA (8)). The daily dose with conv-HC (twice daily regimen, with the higher amount in the morning) was switched to the same of DR-HC: median conv-HC 25 mg (IQR 22.5–37.5) to median DR-HC 25 mg (IQR 20–40, *P* = 0.780). DR-HC was taken once daily, in the morning just after awakening.

Dosages of all other therapies were stable for at least 6 months both before and during the observation period. In PAI patients, fludrocortisone dose was previously adjusted according to blood pressure values and serum potassium levels. Hypothyroid patients were stably treated with levothyroxine to normalize TSH values (around 1–4 mIU/L) in case of primary autoimmune thyroiditis or free thyroxine levels (in the middle quartiles of normal range) in case of secondary hypothyroidism. In hypogonadal patients, adequate substitutive therapy was considered if estradiol levels were in the range for the follicular phase in pre-menopausal-aged women and if total testosterone levels were in the normal range for adult men. Severe growth hormone (GH) deficiency was confirmed by a GH-RH + arginine stimulation test with cut-off limits based upon BMI (13), adequate recombinant human GH therapy was considered if serum IGF1 levels were stably between the 50th and 75th percentile of normal controls matched for sex and age.

After switch to DR-HC, patients were advised to check any initial clinical signs or symptoms of AI (fatigue, nausea, abdominal pain, arterial hypotension).

We also investigated, as a control group, 43 healthy subjects matched for age (45 ± 18 years, *P* = 0.915), gender (15 males and 28 females, *P* = 0.907) and BMI (24.9 ± 3 kg/m^2^, *P* = 0.698). AI was excluded on the basis of normal morning serum cortisol levels (>400 nmol/L^3^). They were all voluntary adults recruited among hospital employees and their family members; none of them were taking exogenous glucocorticoids or drugs that might interfere with the HPA axis; female volunteers were not taking oral or transdermal contraceptives and were investigated in the early follicular phase of the menstrual cycle.

Ethics Committee of Padova University-Hospital approved the study protocol, and all patients gave written informed consent.

### Clinical and biochemical evaluation

At baseline and with DR-HC, routine hematologic and biochemical measurements were performed (sodium, potassium, creatinine, lipid profile), and vital signs were assessed. Wearing light clothing and no shoes, participants were weighed and measured using a balanced beam scale and a vertical ruler: weight was recorded to the nearest 0.5 kg and height to the nearest 0.5 cm, and then BMI was calculated (weight divided by height squared, kg/m^2^). Body surface area (BSA) was calculated with the DuBois and DuBois formula (0.007184 × weight_kg_^0.425^ × height_cm_^0.725^) (14), resulting in a median values of 1.85 m^2^. Waist circumference was measured at the end of natural breath at the midpoint between the top of the iliac crest and the lower margin of the last palpable rib (17). Systolic and diastolic blood pressure (SBP and DBP) were measured on the right arm three times in 5 min with a calibrated standard sphygmomanometer with the appropriate size cuff, after 5 min of resting with patient in a supine position according to the Korotkoff sounds (18).

All patients, irrespective of being PAI or SAI, completed health-related quality of life questionnaire (30-AddiQoL), during conv-HC and with DR-HC. Positive items had scores from 1 to 6, negative statements were reversed (from 6 to 1), and then the scoring was converted to points: 6 = 4 points; 5 and 4 = 3 points; 3 and 2 = 2 points, 1 = 1 point. The algebraic sum of points was calculated: a higher score indicated a better quality of life. The subscale fatigue (8 items: questions 1–5, 23, 26, 27) was also calculated (AddiQoL^fatigue^) (11, 12).

### Salivary cortisol rhythm collection and measurement

Patients collected at home two curves of salivary cortisol, and each one consisted of six samples. Both curves were collected during a normal routine-day, the first during conv-HC (at baseline) and the second at least 6 months after DR-HC (median 8 months, IQR 6–12 months). Samples were collected as previously described in the morning at awakening (F^a^), 1 h and 30 min (F^1.5h^), 6 h (F^6h^, before lunch), 8 h and 30 min (F^8.5h^) and 12 h (F^12h^, before dinner) after the first one, F^b^ was gathered before sleeping (14). F^a^ and F^6h^ samples were collected immediately before taking the morning and afternoon conv-HC dose; F^a^ was gathered before DR-HC. Saliva was collected into cotton-based sampling device with citric acid (Salivette green cap commercial kit, Sarstedt, Numbrecht, Germany). Patients were advised to soak the absorbent cotton for 2 or 3 min, and then the saliva sample was placed in a plastic tube and kept at +4°C. Samples were collected at least 30 min before eating or drinking, to avoid any source of food contamination; patients brushed their teeth at least 30 min before collecting their saliva; smoking or eating licorice was forbidden.

One of the major issue concerning salivary cortisol is home collection (inadequate soaking, blood contamination, wrong sampling time and so on), the protocol was described to the patients in a written form, to ensure a correct home sampling (13, 19).

Salivary F and E were routinely measured by LC-MS/MS with an automated sample preparation, as previously described, utilizing an Agilent HPLC series 1200, with a triple quadrupole mass spectrometer Agilent 6430 equipped with an Electrospray Ionization source in positive ionization mode (Agilent Technologies). The method was linear up to 55.4 and 51 nmol/L, with a low limits quantification of 0.51 and 0.55 nmol/L for F and E, respectively. Within-run and between-run imprecisions were <10%, and the mean recoveries were 101% for F and E (19, 20).

### Statistical analyses

Proportions and rates are calculated for categorical data; continuous data are reported as means and standard error, median and inter-quartile range (IQR) or percentiles (calculated with the National Institute of Standards and Technology formula). Groups were compared by chi-square test for categorical variables and by the Wilcoxon rank-sum test for quantitative variables. Wilcoxon signed-rank test for paired samples was used to compare data at baseline and after GC modification; Kruskal–Wallis Test for Independent Samples was adopted to compare patients and controls.

To assess endogenous daily F exposure, we computed the AUC for salivary F levels at the different time-points respect to the ground (AUC^Fa⟶Fb^) according to the trapezoidal formula (21). Morning (AUC^Fa⟶F6^) and afternoon/evening (AUC^F6⟶Fb^) exposure were evaluated. Linear regression analysis was used to examine the relationship between GC dose or F/E peak and AUC^Fa⟶Fb^ or AUC^Ea⟶Eb^. We performed receiver-operating curve (ROC) analyses to study the sensitivity (SE) and specificity (SP) of salivary F or E; the 95% CI for the AUC was calculated using the Wald approximation, while the binomial method was used for SE and SP. We calculated positive and negative likelihood ratio (LR^pos^ and LR^neg^), with a previously described method (22). Coefficient of variation (CV) was calculated with the formula standard deviation/mean × 100.

The database was managed and statistical analysis performed by SPSS 17 software package for Windows (SPSS, Inc.). Significance level was set as a *P* < 0.05 for all tests.

## Results

Salivary F, E and FEratio levels are depicted in Table 1. F levels were similar in the morning in patients during conv-HC and after switch to DR-HC, but lower in the afternoon/evening with DR-HC than conv-HC. Considering daily F exposure, AUC^Fa→Fb^ was lower with DR-HC despite the same GC dose (25 mg daily). Specifically, morning AUC^Fa→F6h^ levels were similar among conv-HC and DR-HC, contrariwise afternoon/evening F exposure AUC^F6h→Fb^ was lower with DR-HC. F and E levels variability was high: for F 97–140% with conv-HC and 85–130% with DR-HC and for E 70–91% with conv-HC and 66–88% with DR-HC (both similar to controls: F 66–84%, E 32–62%).

**Table 1:** Salivary cortisol (F), cortisone (E), cortisol-to-cortisone ratio (FEratio) levels and daily cortisol exposure (expressed as area under the curve, AUC) in patients with adrenal insufficiency during conventional hydrocortisone (conv-HC) and with dual-release hydrocortisone (DR-HC) compared to healthy controls.

	conv-HC (*n* = 18)	DR-HC (*n* = 18)	*P* conv-HC vs DR-HC	Controls (*n* = 43)	*P* conv-HC vs controls	*P* DR-HC vs controls
F^a^ nmol/L	0.95 (0.5–2.4)	0.5 (0.5–1.2)	0.148	8.2 (5.3–11.6)	<0.001	<0.001
F^1.5h^ nmol/L	35.4 (19.6–83)	34.3 (22.5–72)	0.619	8.2 (4.5–15)	<0.001	<0.001
F^6h^ nmol/L	6.9 (2.4–18.1)	8.3 (4–17.9)	0.925	4.3 (2.3–7.3)	0.164	0.033
F^8.5h^ nmol/L	11.4 (2.2–42.4)	2.7 (1.1–7.3)	0.046	2.8 (1.7–4.4)	0.005	0.752
F^12h^ nmol/L	5.9 (2.8–33.7)	1 (0.5–2.9)	0.002	1.7 (1–3)	<0.001	0.213
F^b^ nmol/L	1.35 (0.5–10.3)	0.6 (0.5–1.4)	0.008	0.9 (0.6–1.2)	0.259	0.204
AUC^Fa→Fb^ nmol∙h/L	12918 (7220–44243)	6364 (4318–17767)	0.045	3645 (2502–5304)	<0.001	0.001
AUC^Fa→F6h^ nmol∙h/L	4710 (2515–11797)	4848 (3147–13422)	0.492	1902 (1170–2598)	<0.001	<0.001
AUC^F6h→Fb^ nmol∙h/L	8391 (2329–23274)	1362 (719–4168)	0.006	1758 (978–2244)	<0.001	0.752
E^a^ nmol/L	1.5 (0.9–4.3)	1.05 (0.5–2.15)	0.249	25 (19.2–29)	<0.001	<0.001
E^1.5h^ nmol/L	31.3 (27.7–77.6)	34.7 (25.9–54.1)	0.679	29.3 (19.4–36)	0.103	0.055
E^6h^ nmol/L	21.9 (10.5–28)	18.15 (12.75–35.76)	0.796	18.3 (14.7–26.3)	0.767	0.936
E^8.5h^ nmol/L	15.7 (6.6–40.8)	7.45 (4.7–21.55)	0.365	13 (11.1–17.8)	0.746	0.061
E^12h^ nmol/L	16.8 (8.8–29.5)	4.15 (1.87–11.32)	0.02	11 (6.8–16.2)	0.055	0.001
E^b^ nmol/L	6.35 (1.8–16.6)	2.35 (0.87–5.5)	0.023	5.4 (4.1–8.7)	0.897	0.003
AUC^Ea→Eb^ nmol∙h/L	17428 (12360–33547)	9235 (7359–23092)	0.044	14277 (11529–17511)	0.042	0.023
FEratio^a^	0.62 (0.34–1)	0.78 (0.3–1.21)	0.778	0.34 (0.27–0.41)	0.005	0.001
FEratio^1.5h^	0.72 (0.35–1.13)	0.81 (0.61–1.06)	0.352	0.3 (0.2–0.4)	<0.001	<0.001
FEratio^6h^	0.34 (0.15–0.47)	0.41 (0.3–0.52)	0.569	0.23 (0.2–0.31)	0.22	<0.001
FEratio^8.5h^	0.49 (0.18–0.90)	0.26 (0.19–0.43)	0.044	0.2 (0.14–0.26)	0.002	0.026
FEratio^12h^	0.5 (0.24–1.14)	0.28 (0.2–0.44)	0.079	0.18 (0.1–0.21)	<0.001	<0.001
FEratio^b^	0.32 (0.2–0.94)	0.46 (0.2–0.72)	0.679	0.18 (0.1–0.2)	0.001	<0.001

Saliva was collected at awakening (F^a^), 1 h and 30 min (F^1.5h^), 6 h (F^6h^), 8 h and 30 min (F^8.5h^) and 12 h (F, before dinner) after the first one, F^b^ was gathered before sleeping. Data are depicted as median and interquartile range (IQR).

Morning F or E levels in AI patients were lower than controls: F^a^ <3 nmol/L presented 90% SE and 98% SP in detecting AI patients (AUC 0.979, 95% CI 0.88–1), achieving LR^pos^ 37.58 (95% CI 5.37–262.19) and LR^neg^ 0.11 (95% CI 0.03–0.4). E^a^ <9.45 nmol/L presented 95% SE and 94% SP to detect AI patients (AUC 0.982, 95% CI 0.89–1), achieving LR^pos^ 18.42 (95% CI 4.75–71.36) and LR^neg^ 0.06 (95% CI 0.01–0.4).

**Figure 1:**
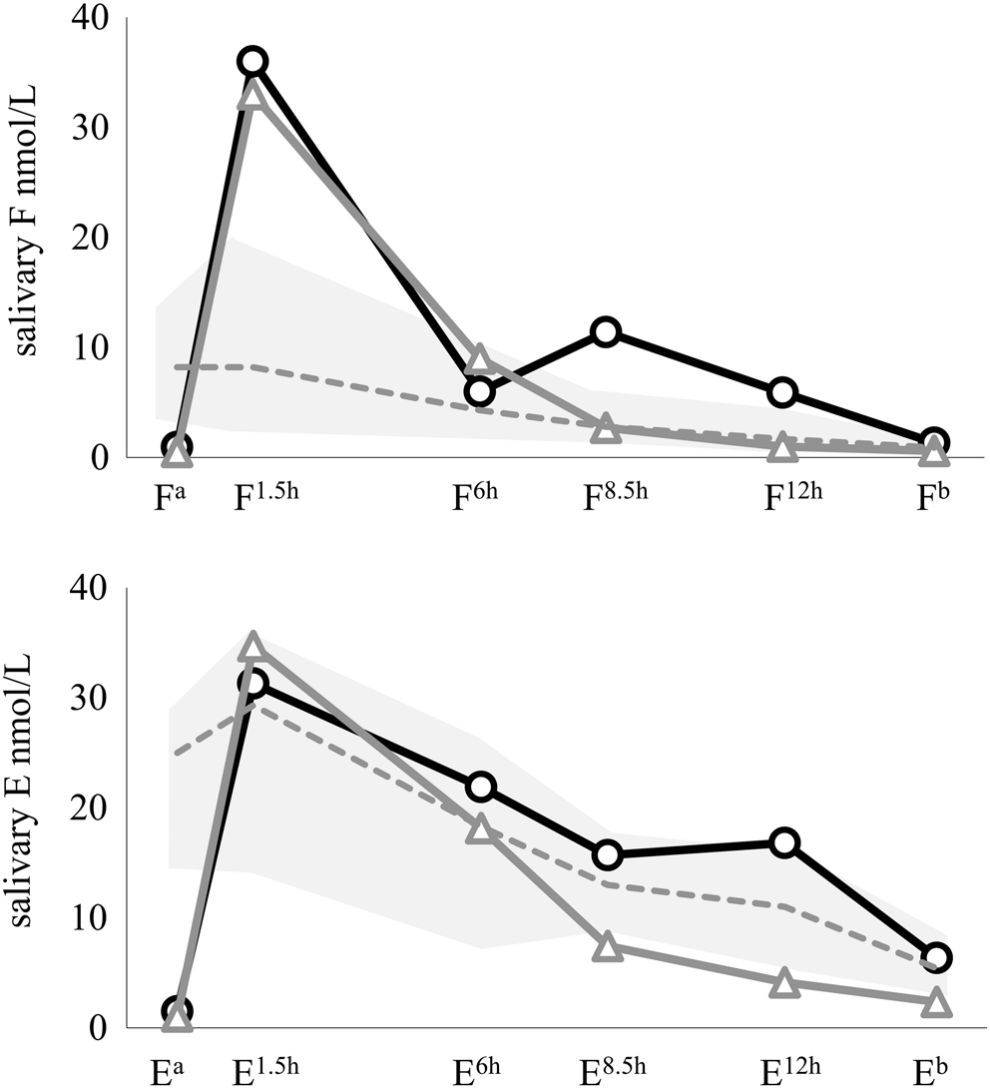
Salivary cortisol (F) and cortisone (E) rhythm in patients with conv-HC (black line) and DR-HC (grey solid line), compared to healthy controls (grey area is included between the 10th and 90th percentile of controls, dotted grey line represents the median).

**Figure 2:**
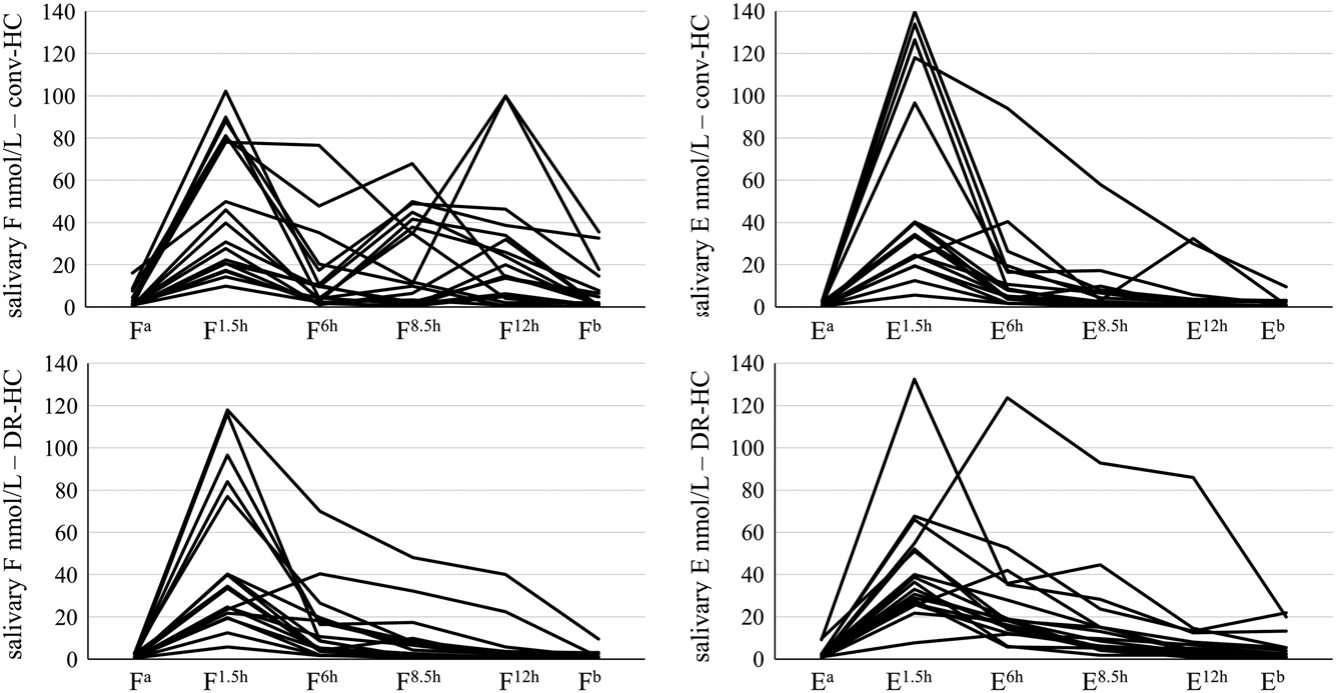
Spaghetti plot of salivary cortisol (F) and cortisone (E) rhythm in patients with conv-HC and DR-HC.

Salivary F levels were higher in patients than in healthy subjects after the GC dose; however, F rhythm in patients with DR-HC was closer to controls (as summarized in Fig. 1, each patient’s curve is depicted in Fig. 2), especially in the afternoon/evening: normalization of evening F exposure (AUC^F6h→Fb^) was observed only in patients with DR-HC. The number of patients with F^b^>2.6 nmol/L (threshold for CS) reduced from to 7/18 with conv-HC to 3/18 with DR-HC (albeit without reaching statistical significance, *P* = 0.072). Considering the 90th percentile of healthy controls’ AUC as the upper limit of normality for F exposure, an increased AUC^Fa→Fb^ was observed in 90% of patients with conv-HC and DR-HC, especially in the morning (both *P* = 1). However, F exposure was increased in the evening only with conv-HC (AUC^F6→Fb^ >90th of controls in 89% with conv-HC and 44% with DR-HC, *P* = 0.005), leading therefore to a normalized F exposure in 56% of AI patients with DR-HC. F^1.5h^ and E^1.5h^ levels correlated with AUC^Fa→F6^ and AUC^Ea→E6^ respectively, with conv-HC as well as DR-HC (adjusted *R* ^2^ 0.742, *P* < 0.001 and adjusted *R* ^2^ 0.716, *P* < 0.001 for F and E with conv-HC and adjusted *R* ^2^ 0.456, *P* = 0.001 and adjusted *R* ^2^ 0.244, *P* = 0.025 for F and E with DR-HC).

E levels in patients during conv-HC were similar to controls (except in the morning), otherwise patients with DR-HC revealed lower E in the afternoon/evening. Increased F levels due to GC contamination were excluded on the basis of normal FEratio in all patients. Clinical data and salivary curves (F, E and FEratio) with baseline HC or CA treatment compared to DR-HC and controls were similar to those obtained in the whole group.

**Figure 3:**
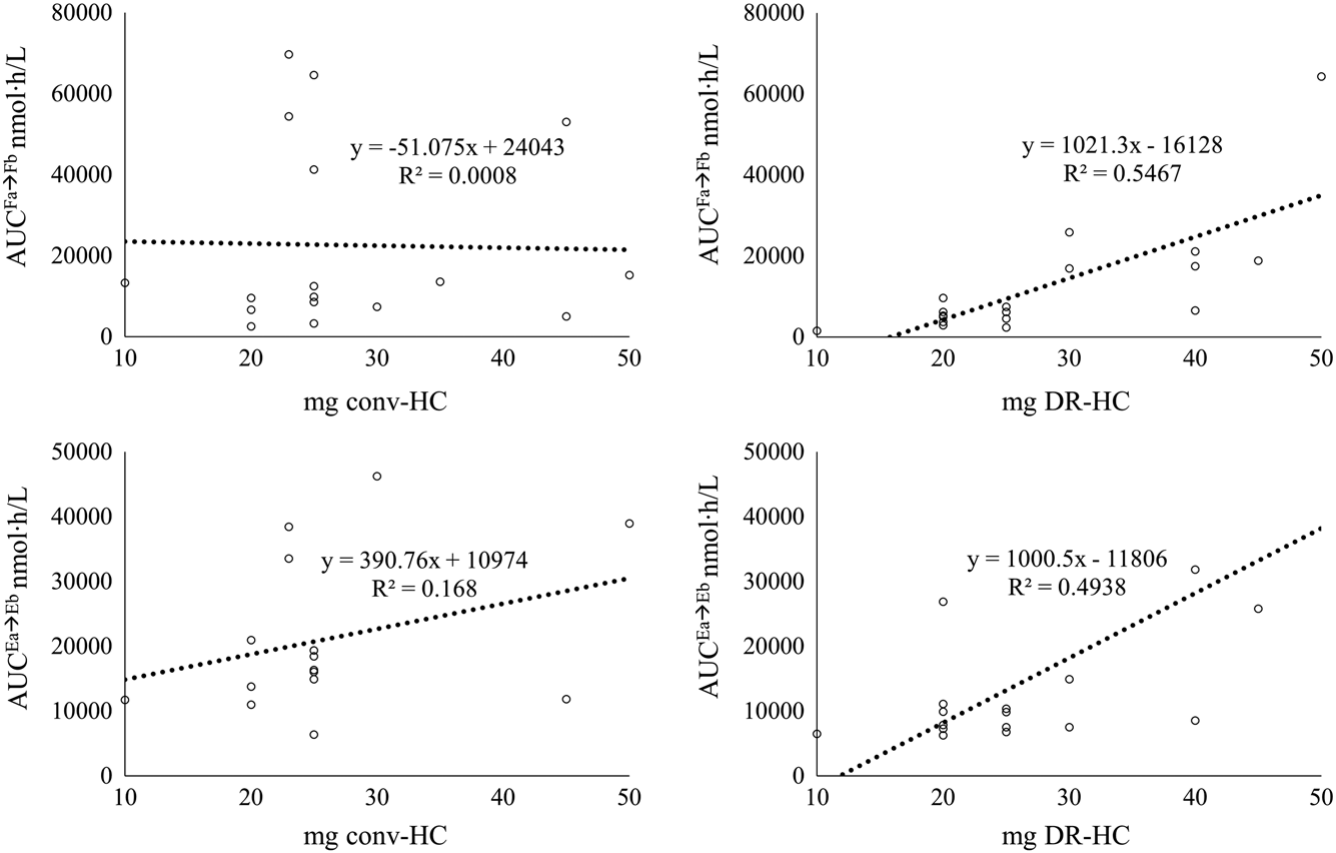
Linear regression (black dotted line) among cortisol or cortisone daily exposure (in term of AUC) and GC dose. Each dot represents a patient.

We correlated daily F or E exposure to GC replacement dose. As reassumed in Fig. 3, we found no correlation among AUC^Fa⟶Fb^ or AUC^Ea⟶Eb^ and conv-HC (respectively adjusted *R* ^2^ −0.061, *P* = 0.886 and adjusted *R* ^2^ 0.103, *P* = 0.121). We observed a correlation among AUC^Fa⟶Fb^ and AUC^Ea⟶Eb^ with DR-HC dose, respectively adjusted *R* ^2^ 0.518 (*P* < 0.001) and adjusted *R* ^2^ 0.494 (*P* = 0.002).

As reported in Table 2, a reduction of total cholesterol levels was observed with DR-HC; HbA1c levels dropped with DR-HC from median 57 mmol/mol (IQR 48–60) to 52 mmol/mol (IQR 44–55, *P* = 0.045) in diabetic patients (*n* = 6). Health-related quality of life questionnaire scores (30-AddiQoL and items of AddiQoL^fatigue^) were similar with conv-HC and DR-HC.

**Table 2:** Clinical and biochemical data at baseline (with conventional hydrocortisone, conv–HC) and after the switch to dual-release hydrocortisone (DR-HC, at least 6 months) in patients with adrenal insufficiency.

	conv-HC (*n* = 18)	DR-HC (*n* = 18)	*P*
Weight (kg)	69.5 (55.2–86.2)	68 (52.5–76.5)	0.694
BMI (kg/m^2^)	24.3 (22.4–30.6)	23.5 (21–26)	0.929
Waist (cm)	92 (74–109)	91.5 (85–96)	0.317
Systolic BP (mmHg)	130 (120–135)	120 (110–130)	0.196
Diastolic BP (mmHg)	82 (80–90)	80 (70–82)	0.453
Na+ (mEq/L)	140 (138–142)	141 (137–142)	0.593
K+ (mEq/L)	4 (3.7–4.3)	4 (3.8–4.3)	0.373
Total cholesterol (mmol/L)	5.99 (4.91–7)	5.48 (4.92–5.6)	0.048
LDL cholesterol (mmol/L)	3.11 (2.82–5.3)	3.02 (2.73–3.67)	0.285
HDL cholesterol (mmol/L)	1.27 (1.14–1.86)	1.57 (1.26–2.03)	0.715
Triglycerides (mmol/L)	1.71 (0.94–2.8)	1.51 (1.1–2.85)	0.963
Glucose (mmol/L)	4.6 (4.34–5.3)	5.2 (4.75–5.95)	0.398
HbA1c (mmol/mol)	39 (34–58)	37 (35–51)	0.27
30-AddiQoL	88 (83–92)	92 (78–95)	0.475
AddiQoL^fatigue^	31 (27.5–32)	33 (28.5–34)	0.386

Data are depicted as median and interquartile range (IQR).

BP, blood pressure.

Salivary F, E and FEratio levels, GC dose with conv-HC or DR-HC, as well as clinical data were similar considering patients with PAI and SAI. Clinical and biochemical data, F or E levels were similar considering HC or CA treatment at baseline.

## Discussion

Substitutive GC treatment in patients with AI represents a challenge in AI (2, 3, 6). None of the GC therapies previously used (HC or CA) were able to mimic perfectly the physiological cortisol circadian rhythm (7), exposing to under- as well as over-treatment (7, 8, 9, 13). Recently, DR-HC has been proposed to patients with AI: its new GC formulation revealed encouraging results in terms of properly mimicking the circadian cortisol profile, closer to normal subjects, reporting a reduction in total daily cortisol exposure, thus leading to an improvement in metabolic and immune parameters (10, 11, 12, 23, 24). We proposed to use salivary cortisol to evaluate the cortisol profile in outpatients with AI, since it is a non-invasive and simple tool to assess the adequacy of GC treatment (13, 14). The use of LC-MS/MS in clinical practice led us to measure F and E (19), enabling the discovery of GC contamination (15, 16). In the present study, we aimed to compare the cortisol daily rhythm and total exposure (considering AUC) in AI patients during conv-HC and after at least 6 months of DR-HC.

Salivary F levels in the morning after conv-HC or DR-HC were similar and higher than controls, as previously described in serum F levels by Johannsson *et al.* (10): we could speculate that the HC dose characterizing the immediate releasing coat is higher than the morning cortisol secretion rate of healthy subjects. Furthermore, salivary F levels may be impacted by the saturation of cortisol-binding globulins (CBG), which are influenced by the distribution of GC dose: it has been previously described that urinary F values were lower when the same GC daily dose was fractioned, thus avoiding high F peak (25, 26). Therefore, we could speculate that the ‘excessive amount’ of free F (unbound to proteins) is excreted with urine, either with conv-HC or with DR-HC. A similar ‘excessive’ F level is observed also with the afternoon dose of conv-HC but not with DR-HC, which presents a cortisol profile closer to controls and a normalization of evening exposure in half subjects. Johannsson *et al*. described that DR-HC reduced 24-h serum GC exposure by nearly 20%, particularly in the afternoon and evening (up to 30 and 60%, respectively (10)). The daily salivary F exposure (AUC^Fa→Fb^) was lower with DR-HC (−50%) despite the same GC dose; the morning AUC^Fa→F6h^ levels were similar after conv-HC or DR-HC: the difference was related to the lower evening and afternoon F exposure (AUC^F6h→Fb^) with DR-HC (−80%, probably due to the bioavailability of the slow-release core). Other authors previously reported a high correlation between serum and salivary F concentrations after substitutive GC treatment (27, 28); therefore, since our aim was to study salivary cortisol, we did not collect a parallel serum profile. The AUC reduction (in serum or saliva) with DR-HC is an unanswered question: Johannsson *et al*. reported that the time to first determined concentration was similar among conv-HC and DR-HC treatment, thus supporting a similar bioavailability considering the two different formulations of HC (10). We could speculate that conv-HC, given in fractionated daily doses to recreate the curve of cortisol rhythm, is associated with considerable variability especially in high F concentrations (as after GC dose), showing several serum cortisol peaks, which might be reflected in elevated serum/salivary F levels and hence an increased cortisol exposure. Actually, the regimens of GC treatment are a critical issue: in recent consensus (6) and guidelines (2) divided daily doses are suggested, the first immediately after waking and the last dose not <6 h before bedtime. Some authors claimed that three are better than two divided doses (especially HC with 2:1:1, 3:1:1, 3:2:1 and so on regimens (6)). Contrariwise, in a large survey, a larger use of twice-daily regimen is reported (29): further studies are needed to establish the cortisol exposure during different regimens and formulations of GC treatment. In our clinical practice, the cortisol curve is evaluated through six daily salivary samples with LC-MS/MS. The variability of salivary F and E is high, we acknowledge that a protocol with an increased number of salivary samples could be able to detect all the cortisol fluctuations; however, both cost-effectiveness and patients’ compliance are crucial in clinical practice. Moreover, subjects with AI are motivated to supervise their GC treatment, thus increasing their compliance. We could also suggest a simplified assessment based on the second salivary collection after the morning dose of conv-HC or DR-HC, due to the correlation between salivary F or E levels and respectively AUC, as previously described (13).

It is well known that increased evening cortisol levels may contribute to metabolic consequences such as impaired glucose metabolism, as observed in patients with CS (30, 31). In this study, we confirmed the reduction of total cholesterol and HbA1c in diabetic subjects, as previously reported (10, 12). Therefore, the reduction of GC exposure in the afternoon and evening with DR-HC may positively reduce long-term consequences. In our clinical practice, we put an effort to reduce GC treatment to modern ‘low doses’, closer to the daily cortisol-rate production based upon BSA: one of the major drawback related to GC treatment is the lack of objective methods to reveal under- or over-replacement (1, 2, 3, 6). Therefore, identifying the lowest GC dose that relieves symptoms of insufficiency, while avoiding cortisol excess, is a challenge.

We did not observe a self-perceived quality of life amelioration using health-related questionnaire scores (both 30-AddiQoL and AddiQoL^fatigue^), as previously reported (11). On the contrary, larger (24) or longer (12) studies reported an improvement of quality of life with DR-HC: in our study, size and duration were not sufficient.

Recently, it has been proposed that salivary E reflects serum F profile, enabling its noninvasive measurement (15). Low levels of F or E presented high diagnostic accuracy to discover AI: SE and SP were >90% with a respective threshold of <3 nmol/L and <9.45 nmol/L. We previously described similar data (32), however, both Deutschbein *et al*. (33) and Restituto *et al*. (34) have reported a lower diagnostic accuracy: they both used immunoassay, thus suffering of cross-reaction among F and E. Recently, a superior diagnostic accuracy of E has been described for cortisol-related disorders (35); however, in our study the likelihood ratios of F and E were similar. Diagnosis of SAI is a challenge and a matter of clinical debate. Endocrine Society’s guidelines suggested to measure serum cortisol after ACTH test (3); nevertheless, HPA axis could recover after injury (i.e. after pituitary surgery (36)): in this setting, salivary F or E could be used in a planned follow-up in order to perform the dynamic test at the correct time, i.e. when morning F or E levels start to decrease. We observed that salivary E levels and rhythm in patients after conv-HC were similar to controls, otherwise patients with DR-HC revealed lower E levels in the afternoon/evening. Debono *et al*. reported that salivary F post-oral HC produced spurious results, probably due to contamination, whereas salivary E correlated strongly with serum cortisol (35). We did not measure serum cortisol in our patients; therefore, we could not establish which steroid allows a better estimation of GC treatment in AI. The measurement of oral GC contamination is not a routine procedure in clinical practice: in 2012, Raff *et al*. proposed normal E level and increased FEratio (<1.2) (15). Solid reports regarding GC contamination are lacking in literature, and we have not performed contamination tests. FEratio was higher in patients than in controls (irrespective of treatment with conv-HC or DR-HC): we could speculate that the reduction of E levels in AI patients could explain their increased salivary FEratio, because they used HC (which is F) or CA (activated to F in the liver). These ‘normal-increased’ F levels could be combined with a reduced activity of 11β-hydroxysteroid dehydrogenase type 2 in salivary glands in patients with AI, probably secondary to the enzyme saturation, as that observed in case of increased F levels in patients with CS (37, 38). We correlated daily F and E exposure to GC replacement dose: we found no correlation among AUC^Fa⟶Fb^ or AUC^Ea⟶Eb^ with conv-HC, as previously reported considering CA (13) and recently remarked by Ross and colleagues (39) (using LC-MS/MS). Contrariwise, we observed a positive correlation between AUC^Fa⟶Fb^ and AUC^Ea⟶Eb^ with DR-HC dose, more related to its peculiar formulation and pharmacokinetics rather than its dose (being it the same of conv-HC). The interest regarding E, especially in saliva, is rising in recent literature. Considering AI, measuring salivary E after HC does not have the same risk for drug contamination as observed when measuring salivary F (15); moreover, salivary E levels are irrespective of CBG levels (35). Concerning the diagnosis of CS, salivary E after dexamethasone suppression test resulted in an increased diagnostic accuracy (40). On the other hands, E measurement is limited to third-care hospital or referral centers. To conclude, despite promising data, the clinical utility of salivary F or E as a marker for the monitoring of patients with AI on GC replacement therapy is still uncertain, and further studies are needed to ascertain the role of routine measurement of steroids with LC-MS/MS.

Beside strengths, our study presents some limitations. First, the design of the study (observational, open and not randomized); patients were not blinded to the treatment and cohort of subjects was not large, resulting from the high specific selection criteria established. Secondly, before enrollment patients were treated with different GC (HC or CA); therefore, the pharmacokinetic results obtained (mainly AUC) have to be confirmed in larger series of patients treated with one compound. To obtain solid data, we used anti-inflammatory equivalents, as reported in the Endocrine Society’s guidelines (3); other equivalent doses are proposed, as the anti-inflammatory equivalents or growth-retarding cortisol equivalents (41, 42); however, they are not used worldwide. Furthermore, also a three times daily dosing with conv-HC could improve daily cortisol curve; however, we decided to consider only patients with twice daily GC dosing, to ensure appropriate comparison among HC and CA. Follow-up lasts less than 1 year, probably insufficient to point out long-term improvements.

To conclude, salivary cortisol could be a marker to assess the cortisol profile in patients with DR-HC and might provide new insights in the study of patients with AI.

## Declaration of interest

The authors declare that there is no conflict of interest that could be perceived as prejudicing the impartiality of the research reported.

## Funding

This study did not receive any specific grant from any funding agency in the public, commercial or not-for-profit sector.
